# Cationic nanoparticles for delivery of amphotericin B: preparation, characterization and activity *in vitro*

**DOI:** 10.1186/1477-3155-6-6

**Published:** 2008-05-07

**Authors:** Débora B Vieira, Ana M Carmona-Ribeiro

**Affiliations:** 1 Departamento de Bioquímica, Instituto de Química, Universidade de São Paulo, CP 26077, CEP 05513-970, São Paulo, Brazil

## Abstract

**Background:**

Particulate systems are well known to be able to deliver drugs with high efficiency and fewer adverse side effects, possibly by endocytosis of the drug carriers. On the other hand, cationic compounds and assemblies exhibit a general antimicrobial action. In this work, cationic nanoparticles built from drug, cationic lipid and polyelectrolytes are shown to be excellent and active carriers of amphotericin B against *C. albicans*.

**Results:**

Assemblies of amphotericin B and cationic lipid at extreme drug to lipid molar ratios were wrapped by polyelectrolytes forming cationic nanoparticles of high colloid stability and fungicidal activity against *Candida albicans*. Experimental strategy involved dynamic light scattering for particle sizing, zeta-potential analysis, colloid stability, determination of AmB aggregation state by optical spectra and determination of activity against *Candida albicans *in vitro from cfu countings.

**Conclusion:**

Novel and effective cationic particles delivered amphotericin B to *C. albicans *in vitro with optimal efficiency seldom achieved from drug, cationic lipid or cationic polyelectrolyte in separate. The multiple assembly of antibiotic, cationic lipid and cationic polyelctrolyte, consecutively nanostructured in each particle produced a strategical and effective attack against the fungus cells.

## Background

In the recent years, much work has been devoted to characterize nanoparticles and their biological effects and applications. These include bottom-up and molecular self-assembly, biological effects of naked nanoparticles and nano-safety, drug encapsulation and nanotherapeutics, and novel nanoparticles for use in microscopy, imaging and diagnostics [1]. Particulate drug delivery systems such as polymeric microspheres [2], nanoparticles [3,4], liposomes [5,6], and solid lipid nanoparticles (SLNs) [7] offer great promise to achieve the goal of improving drug accumulation inside cancer cells without causing side effects. Particulate systems are well known to be able to deliver drugs with higher efficiency with fewer adverse side effects [6,8]. A possible mechanism is increase of cellular drug uptake by endocytosis of the drug carriers [9-11]. The emergence of the newer forms of SLN such as polymer-lipid hybrid nanoparticles, nanostructured lipid carriers and long-circulating SLN may further expand the role of this versatile drug carrier aiming at chemotherapy with cancer drugs [12]. Recently, new nanoparticulate delivery systems for amphotericin B (AmB) have been developed by means of the polyelectrolyte complexation technique [13,14]. Two oppositelly charged polymers were used to form nanoparticles through electrostatic interaction as usual for the Layer-by-Layer approach (LbL). This approach creates homogeneous ultrathin films on solid supports based on the electrostatic attraction between opposite charges [15]. Consecutively alternating adsorption of anionic and cationic polyelectrolytes or amphiphiles from their aqueous solution leads to the formation of multilayer assemblies [16].

On the other hand, some double-chained synthetic lipids such as dioctadecyldimethylammonium bromide (DODAB) or sodium dihexadecylphosphate (DHP) self-assemble in aqueous solution yielding closed bilayers (vesicles) or disrupted vesicles (bilayer fragments, BF, or disks) depending on the procedure used for dispersing the lipid [17]. DODAB, in particular, bears a quaternary ammonium moiety as cationic polar head, which imparts to this cationic lipid outstanding anti-infective properties [18]. Both amphotericin B and miconazole self-assemble and solubilize at hydrophobic sites of DODAB or DHP bilayer fragments in water solution exhibiting *in vivo *therapeutic activity [19-22]. Over the last decade, our group has been describing the anti-infective properties of cationic bilayers composed of the synthetic lipid dioctadecyldimethyl ammonium bromide (DODAB) [17,18,21-27]. Adsorption of DODAB cationic bilayers onto bacterial cells changes the sign of the cell surface potential from negative to positive with a clear relationship between positive charge on bacterial cells and cell death [26]. Regarding the mechanism of DODAB action, neither bacterial cell lysis nor DODAB vesicle disruption takes place [27]. Recently, it was shown that the critical phenomenon determining antifungal effect of cationic surfactants and lipids is not cell lysis but rather the reversal of cell surface charge from negative to positive [28]. In this work, we combine the SLN and the LbL approaches to develop novel and effective cationic particles to deliver AmB to *C. albicans*. Cationic microbicides self-assemble in a single supramolecular structure. The first attack against the fungus comes from an outer cationic polyelectrolyte layer. Thereafter the inert carboxymethylcellulose (CMC) layer is unwrapped so that monomeric AmB solubilized at the edges of DODAB bilayer fragments (BF) and the BF themselves can contact the fungus cell. Maybe this design represents a very effective cocktail against multidrug resistance. Complete loss of fungus viability could not be achieved before at the same separate doses of each component.

## Results and Discussion

### Colloid stability and antifungal activity of cationic bilayer fragments/amphotericin B/carboxymethyl cellulose/poly(diallyldimethylammonium) chloride at low drug-to-lipid molar proportion

Chemical structures of amphotericin B (AmB), carboxymethylcellulose (CMC), poly(diallyldimethylammonium chloride) (PDDA) and the cationic lipid dioctadecyldimethylammonium bromide (DODAB) are on Table 1. DODAB self-assembly in water dispersion yields bilayer fragments (BF) by ultrasonic input with a macrotip probe.

**Table 1:** Sizing and zeta-potential of drug, cationic lipid and anionic polyelectrolyte in separate or as assemblies

Dispersion	[DODAB] (mM)	[AmB] (mM)	[CMC] (mg/mL)	D ± δ (nm)	ζ ± δ (mV)
AmB in water	---	0.005	---	433 ± 5	-26 ± 3
DODAB BF	1	- - -	---	79 ± 2	41 ± 2
DODAB BF/AmB	1	0.005	---	79 ± 1	42 ± 2
DODAB BF/AnB/CMC	1	0.005	0.01	88 ± 1	40 ± 1
	1	0.005	0.1	145 ± 1	32 ± 2
	1	0.005	1	90 ± 2	-50 ± 2
					
AmB in water	---	0.050	---	360 ± 4	-26 ± 3
AmB in IGP	---	0.050	---	75 ± 2	-27 ± 1
DODAB BF in IGP	0.1	- - -	---	75 ± 1	40 ± 1
AmB/DODAB BF	0.1	0.050	---	195 ± 3	9 ± 1
AmB/DODAB BF/CMC	0.1	0.050	0.001	199 ± 1	16 ± 1
	0.1	0.050	0.01	1280 ± 80	4 ± 1
	0.1	0.050	0.1	230 ± 2	-34 ± 1

Zeta-average diameter (D) and zeta-potentials (ζ) for different dispersions aiming at formulation of AmB in cationic lipid DODAB and CMC. Dispersions were prepared either in Milli-Q water or in IGP buffer.

The existence of bilayer fragments from synthetic lipids such as sodium dihexadecylphosphate, or dioctadecyldimethylammonium bromide or chloride obtained by sonication with tip has been supported by the following evidences: (i) osmotic non-responsiveness of the dispersion indicative of absence of inner vesicle compartment [29]; (ii) TEM micrographs with electronic staining [30]; (iii) cryo-TEM micrographs [31]; (iv) fluid and solid state coexistence and complex formation with oppositely charged surfactant [32]; (v) solubilization of hydrophobic drugs at the borders of DODAB bilayer fragments, which does not occur for DODAB closed bilayer vesicles [19-21,33,34]. They differ from the closed vesicles by providing hydrophobic borders at their edges that are absent in closed bilayer systems such as vesicles or liposomes. Under conditions of low ionic strength, due to electrostatic repulsion, the charged bilayer fragments remain colloidally stable in aqueous dispersions [19-21,33,34].

**Figure 1:**
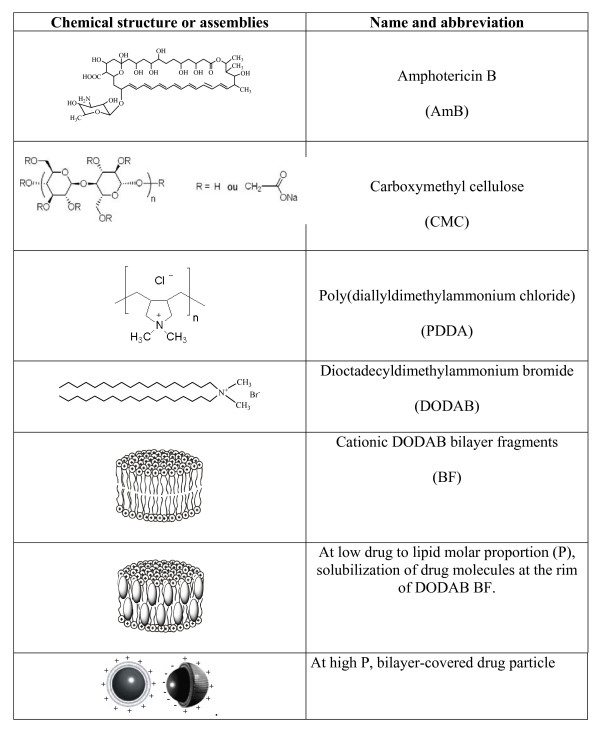
**Chemical structure or schematic assemblies of compounds used to formulate amphotericin B**. Each molecule of amphotericin B that was solubilized at the edges of DODAB bilayer fragments was represented by an ellipsoid whereas aggregated drug forming a particle was represented by solid spheres.

In fact, DODAB BF have been used to solubilize AmB [19] at room temperature as schematically shown in Figure 1. This solubilization takes place at low drug-to-lipid molar proportions (low P) and presents certain limitations: 1) hydrophobic edges of bilayer fragments have a limited capacity of solubilizing the hydrophobic drug; 2) the bilayer core in the rigid gel state is too rigid to allow solubilization of AmB at room temperature being a poor solubilizer for this difficult, hydrophobic drug [19,20,33,35]. On the other hand, at high P, AmB aggregates in water solution can be considered as drug particles. These can be surrounded by a thin cationic DODAB bilayer as previously described [35] (Figure 1).

The physical properties of different dispersions such as size and zeta-potential are given in Table 1 both at low and high P. The drug in water exhibits substancial aggregation (Dz = 360–433 nm), as expected from its hydrophobic character. The drug particle presents a negative zeta-potential of -26 mV explained by dissociation of its carboxylate moiety at the pH of water [35]. Upon changing the medium to IGP buffer, as previously reported, a decrease in size for AmB aggregates was observed (Dz = 75 nm) (Table 1), due to the chaotropic (dispersing) effect of dihydrogenphosphate anion on AmB aggregates [35]. Both types of AmB aggregates interacted with DODAB BF yielding either loaded BF fragments at low P or DODAB covered drug particles at high P. The characteristics of these cationic assemblies before and after their interaction with oppositely charged CMC over a range of concentrations (0.001–1.0 mg/mL) are in Table 1. At low P, charge reversal took place above 1 mg/mL CMC whereas at high P, it occurred above 0.1 mg/mL CMC (Table 1).

**Figure 2:**
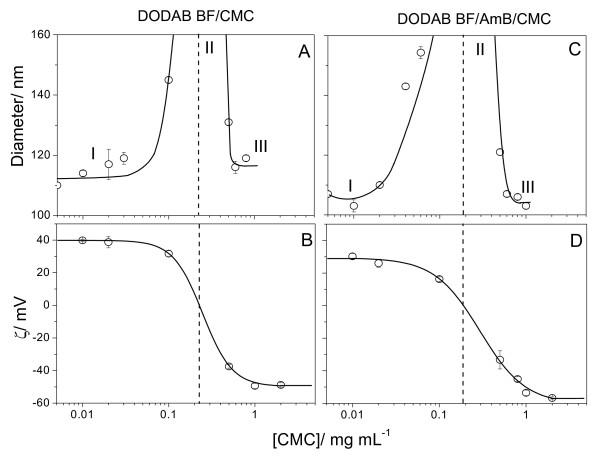
**Amphotericin B solubilized in cationic bilayer fragments adsorbs a layer of carboxymethyl cellulose**. Effect of CMC concentration on zeta-average diameter (A, C) and zeta-potential (B, D) of unloaded DODAB BF (A, B) or DODAB BF/AmB (C, D) at low drug-to-lipid molar proportion. Final DODAB and/or AmB concentrations are 1 and 0.005 mM, respectively. The three different moieties of the curves were named I, II and III corresponding to positive, zero and negative zeta-potentials, respectively.

At low P, the effect of CMC concentration on DODAB BF/CMC (unloaded control) or DODAB BF/AmB/CMC properties is in Figure 2. At low P and 1 mg/mL CMC, DODAB BF/AmB/CMC anionic complexes present 90 nm mean diameter and -50 mV of zeta-potential. The low size and large surface potential mean high colloid stability, so that this was the condition chosen for coverage with cationic polyelectrolytes. In the presence of CMC, there are two regions of colloid stability for cationic or anionic assemblies characterized by small sizes: regions I and III, and one region of instability: region II, characterized by aggregation and large sizes (Figure 1). Charged particles covered by oppositely charged polyelectrolytes exhibited similar profiles for the colloid stability as a function of polyelectrolyte concentration [36,37].

**Figure 3:**
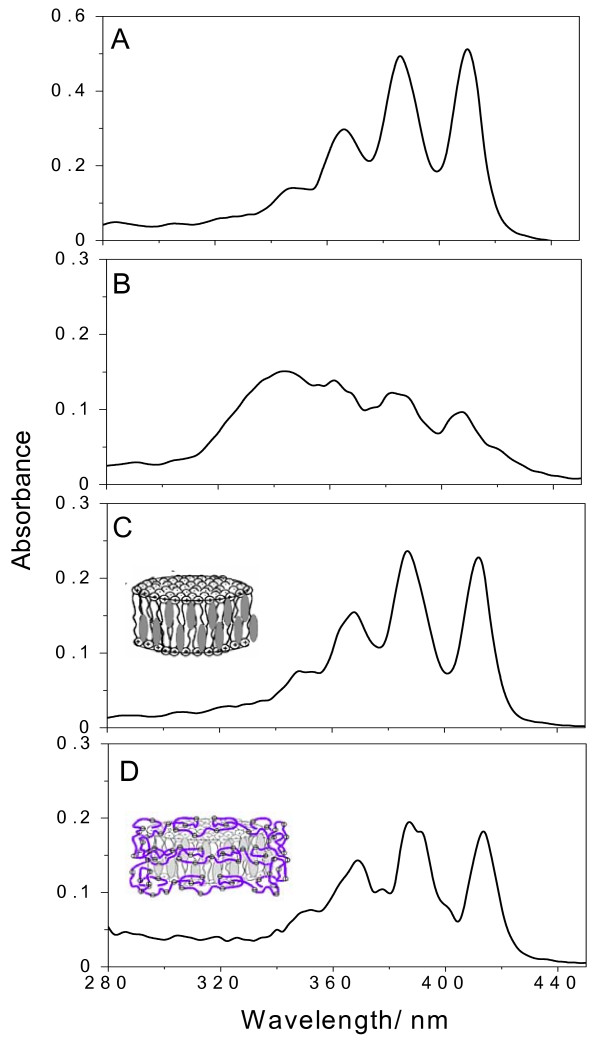
**Adsorption of carboxy methylcellulose onto amphotericin B- cationic lipid assemblies preserves monomeric state of the drug at the edges of cationic bilayer fragments**. Optical spectra of AmB in: 1:1 DMSO: methanol (best organic solvent mixture) (A); water (B); DODAB BF (C) or DODAB BF/AmB/CMC complexes (D). Final DODAB, AmB and/or CMC concentration are 1 mM, 0.005 mM and 1 mg.mL-1, respectively.

The aggregation state of AmB at low P was evaluated from optical spectra (Figure 3). The drug in DMSO:methanol 1:1 yields a spectrum of completely solubilized, nonaggregated drug since this organic solvent mixture is the one of choice for AmB solubilization (Figure 3A). The drug in water exhibits the typical spectrum of aggregated AmB (Figure 3B). As depicted from AmB spectrum in DODAB BF (Figure 3C) or DODAB BF/AmB/CMC (Figure 3D), the drug is found in its monomeric state and completely solubilized. In fact, solubilization of AmB in DODAB BF, at low P, was previously described [19]. This formulation employing DODAB BF at low P was very effective *in vivo *[21] and exhibited low nephrotoxicity [22].

**Figure 4:**
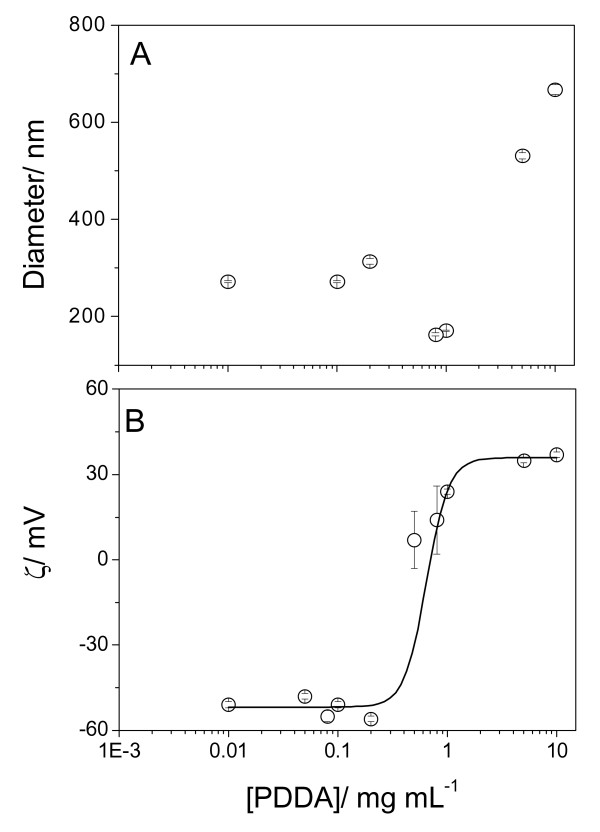
**Adsorption of poly(diallyldimethylammonium) chloride onto carboxy methyl cellulose layer of amphotericin B- cationic bilayer fragment**. Effect of PDDA concentration on z-average diameter (A) and zeta-potential (B) for DODAB BF/AmB/CMC/PDDA assemblies. Final DODAB, AmB and CMC concentrations were 1 mM, 0.005 mM and 1 mg.mL^-1^, respectively. Interaction time between DODAB BF/AmB and CMC is 20 minutes. Thereafter, the interaction between DODAB BF/AmB/CMC and PDDA lasted 30 minutes.

**Figure 5:**
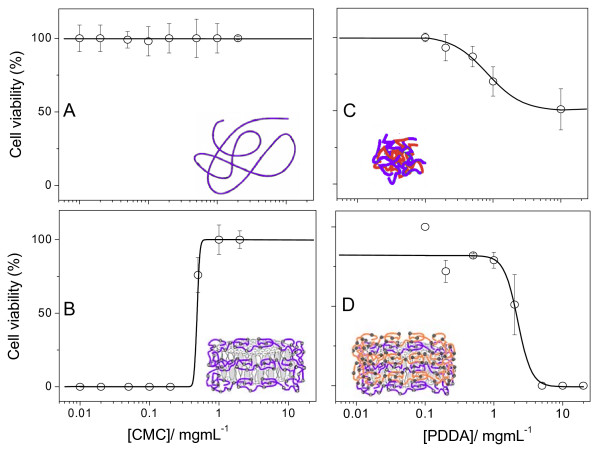
**Fungicidal activity of different assemblies at low P against fungus**. Cell viability (%) of *Candida albicans *(1 × 10^6 ^cfu/mL) as a function of polyelectrolytes concentration. Cells and CMC (A); DODAB/AmB/CMC (B); CMC/PDDA (C) and DODAB/AmB/CMC/PDDA (D) interacted for 1 h before dilution and plating on agar of 0.1 mL of the diluted mixture (1:1000 dilution).

At low P, the effect of [PDDA] on sizes and zeta-potentials of DODAB BF/AmB/CMC assemblies at 1 mM DODAB, 0.005 mM AmB and 1 mg/mL CMC is on Figure 4. The region of PDDA concentrations for size minimization and high colloid stability was very narrow and around 1 mg/mL PDDA. Below and above this concentration, about 300 nm and negative zeta-potentials, or 500–700 nm of zeta-average diameter and positive zeta-potentials were obtained, respectively (Figure 4). Size minimization at Dz = 171 nm and zeta-potential = 24 mV for the DODAB BF/AmB/CMC/PDDA assembly was not related to optimal fungicidal activity as depicted from the 79% of *C. albicans *viability (Table 2). Possibly, the total positive charge on the assembly was not sufficient to substantially reduce fungus viability. For final coverage with polylysines (PL) of increasing molecular weight at 1 mg/mL PL, there was an increase in the final zeta-potential modulus and a larger loss of viability (Table 2). The DODAB BF/AmB/CMC/PDDA formulation at low P was 100% effective against the fungus only at 5 mg/mL PDDA (Figure 5D).

**Table 2:** Sizing, zeta-potential and antifungal activity of drug, cationic lipid, and polyelectrolyte(s) assemblies

Cationic lipid, drug and polyelectrolyte assemblies	D ± δ (nm)	ζ ± δ (mV)	Viability (%)
DODAB BF (0.6)/AmB (0.005)/CMC (1)/PDDA(1)	171 ± 1	24 ± 2	79 ± 5
DODAB BF (0.6)/AmB (0.005)/CMC (1)/PL_5000–10000 _(1)	92 ± 4	40 ± 1	71 ± 4
DODAB BF (0.6)/AmB (0.005)/CMC (1)/PL_30000–70000 _(1)	138 ± 5	50 ± 3	21 ± 9
DODAB BF (0.6)/AmB (0.005)/CMC (1)/PL_70000–150000 _(1)	148 ± 5	60 ± 3	13 ± 5
AmB (0.05)/DODAB BF (0.06)/CMC (0.1)/PDDA (1)	280 ± 2	35 ± 1	27 ± 2
AmB (0.05)/DODAB BF (0.06)/CMC (0.1)/PL_5000–10000 _(1)	238 ± 1	25 ± 7	37 ± 1
AmB (0.05)/DODAB BF (0.06)/CMC (0.1)/PL_30000–70000 _(1)	326 ± 5	36 ± 3	23 ± 6
AmB (0.05)/DODAB BF (0.06)/CMC (0.1)/PL_70000–150000 _(1)	417 ± 3	47 ± 5	11 ± 3

Zeta-average diameter (D) and zeta-potential (ζ) of novel cationic AmB formulations and their effect on *C. albicans *viability at low and high drug-to-lipid molar proportions. Concentrations are given within parentheses in mg/mL. One should notice that polylysine (PL) with diferent molecular weights may alternatively replace PDDA and be used to control the positive zeta-potential of the outer layer.

The importance of large positive zeta-potentials for high efficiency of drug assemblies with DODAB BF and polyelectrolytes can be clearly seen from Figure 5. Negatively charged assemblies like those in Figure 5A and 5B yielded 100% of cell viability. Positively charged assemblies obtained upon increasing [PDDA] reduced cell viability to 50% (CMC/PDDA) (Figure 5C) or to 0% (DODAB BF/AmB/CMC/PDDA above 5 mg/mL PDDA) (Figure 5D). The schematic drawing in Figure 5D illustrates the layered assembly of microbicides in a single supramolecular assembly. The first attack comes from the outer cationic polyelectrolyte layer. Upon unwrapping this first layer and the second inert CMC layer, monomeric AmB contacts the fungus cell followed by the also effective DODAB action. Maybe this design represents a very effective assembly against multidrug resistance. Complete loss of fungus viability can seldom be achieved at the same separate doses of each component [25].

### Colloid stability and antifungal activity of AmB/DODAB BF/CMC/PDDA at high P

**Figure 6:**
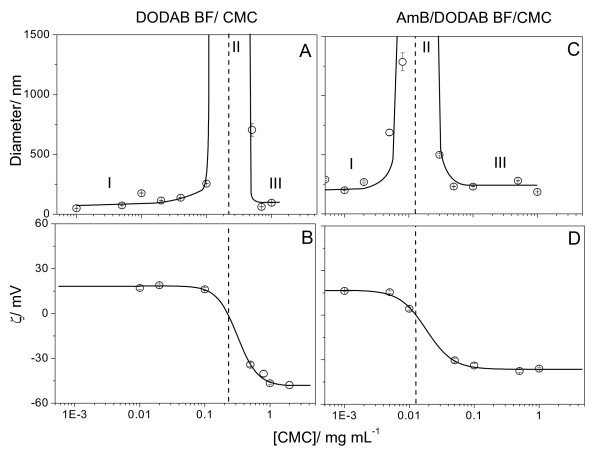
**Amphotericin B aggregates covered by a layer of cationic lipid adsorb a layer of carboxymethyl cellulose**. Effect of CMC concentration on zeta-average diameter (A, C) and zeta-potential (B, D) of DODAB BF (A, B) or AmB/DODAB BF (C, D) at high P. Final DODAB and/or AmB concentrations were 0.1 and 0.05 mM, respectively. The three different moieties of the curves were named I, II and III corresponding to positive, zero and negative zeta-potentials, respectively. Interactions DODAB BF/CMC or AmB/DODAB BF/CMC took place over 20 minutes before measurements. One should notice that, at high P, [DODAB] concentration is 20 times smaller than at low P (Figure 1) surrounding drug aggregates as a thin layer of cationic lipid. ^34^

The complexation between DODAB BF and CMC was previously studied in detail by our group [36]. DODAB BF at 0.1 mM DODAB and CMC (0.001–2 mg/mL) are, in fact, electrostatically driven to complexation from the electrostatic attraction (Figure 6A and 6B).

At high P, 0.1 mM DODAB BF is sufficient to cover all AmB particles present in dispersion at 0.05 mM AmB with a thin, possibly bilayered, 6–8 nm DODAB cationic shell as previously described [35]. This cationic interface is expected to interact with the oppositely charged CMC polyelectrolyte. At 0.1 mg/mL CMC, AmB/DODAB BF/CMC anionic complexes present high colloid stability, 230 nm mean diameter and -34 mV of zeta-potential (Figure 6C and 6D). This condition was chosen for further coverage with cationic polylectrolytes.

**Figure 7:**
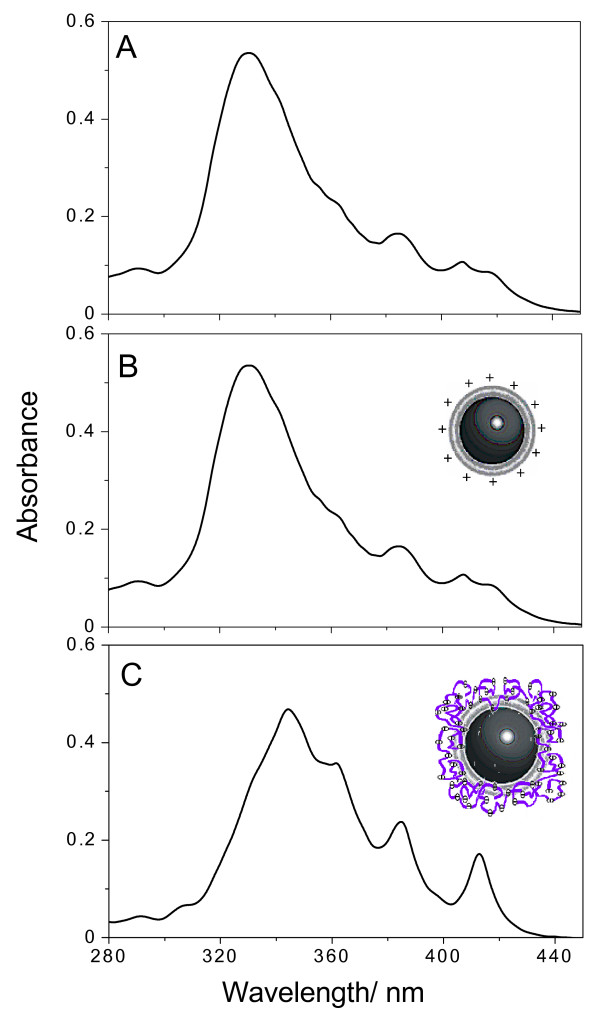
**Amphotericin B is found as in the aggregated state (drug particles) covered by a thin layer of cationic lipid further surrounded by a layer of carboxymethyl cellulose at high P**. Optical spectra of AmB in isotonic glucose buffer (A); AmB/DODAB BF (B) or AmB/DODAB/CMC complexes (C). Final DODAB, AmB and/or CMC concentration were 0.1 mM, 0.05 mM e 0.1 mg.mL^-1^, respectively. These conditions yield complexes at high P.

Regarding the aggregation state of AmB, as expected, at 0.05 mM AmB, the majority of drug molecules were found in the aggregated state. Spectra in IGP buffer (Figure 7A), after drug particle coverage with 0.1 mM DODAB BF (Figure 7B) or with 0.1 mM DODAB BF plus 0.1 mg/mL CMC (Figure 7C) revealed the typical profile of aggregated drug. The spectrum in Figure 7C indicates a certain amount of monomeric drug not present in the other spectra (Figure 7A and 7B). Possibly, CMC sterically stabilized DODAB BF preserving hydrophobic sites of DODAB BF to be occupied by the monomeric drug. In absence of CMC, DODAB BF might fuse diminishing drug solubilization at their rim.

**Figure 8:**
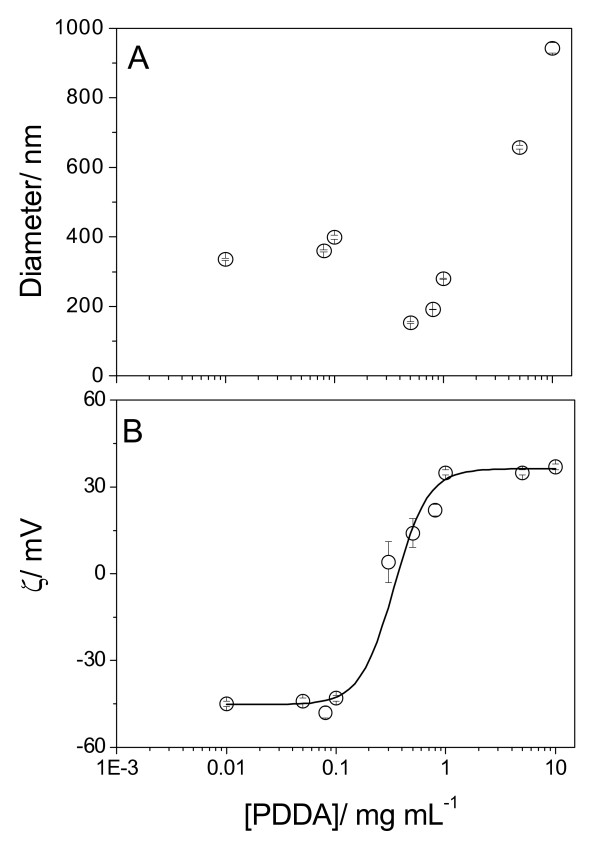
**Amphotericin B particles covered by a thin layer of cationic lipid, at high P, and surrounded by a layer of carboxymethyl cellulose further adsorb a layer of cationic polyelectrolyte**. Effect of PDDA concentration on zeta-average diameter (A) and zeta-potential (B) for AmB/DODAB BF/CMC/PDDA complexes. Final DODAB, AmB and CMC concentrations were 0.1 mM, 0.05 mM and 0.1 g.L^-1^, respectively. Interactions DODAB BF/AmB and CMC took place over 20 minutes and AmB/DODAB BF/CMC and PDDA, over 30 minutes.

At the chosen condition for the AmB/DODAB BF/CMC assembly, the effect of increasing [PDDA] was an initial colloid stabilization (decrease in size) around 1 mg/mL PDDA followed by further destabilization (increase in size) above this concentration (Figure 8A), possibly due to bridging flocculation [38]. Zeta-potential displayed the usual sigmoidal dependence on [PDDA] (Figure 8B).

**Figure 9:**
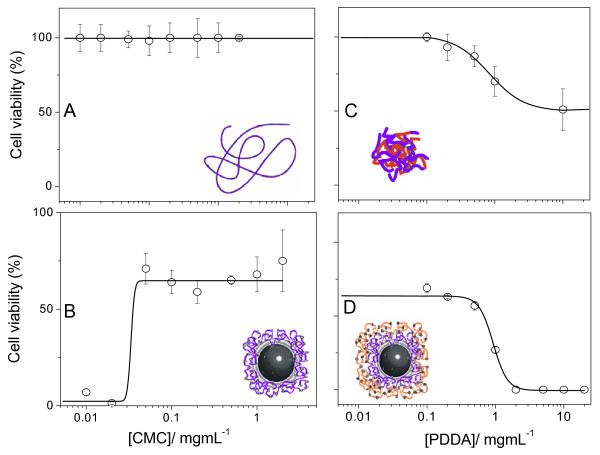
**Fungicidal activity of different assemblies at high P against fungus**. Cell viability (%) of *Candida albicans *(1 × 10^6 ^cfu/mL) as a function of CMC (A, B) or PDDA (C, D) concentration in the presence of different assemblies: CMC only (A); AmB/DODAB/CMC (B); CMC/PDDA (C) and AmB/DODAB/CMC/PDDA (D). The assemblies interacted with cells for 1 h before dilution (1:1000) and plating on agar of 0.1 mL of the diluted mixture.

The importance of positively charged assemblies at high P for fungicidal activity is emphasized in Figure 9. *C. albicans *remains 100% viable in the presence of negatively charged CMC only (Figure 9A), 70% viable in the presence of negatively charged AmB/DODAB BF/CMC at high P (Figure 9B), 50–60% viable in the presence of CMC/PDDA at [PDDA] > 1 mg/mL and 0% viable in the presence of AmB/DODAB BF/CMC/PDDA at PDDA ≥ 2 mg/mL (Figure 9D).

**Figure 10:**
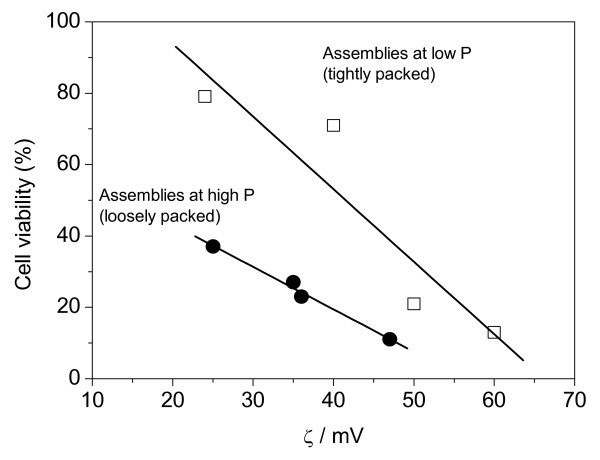
**Correlation between Candida albicans viability (%) and zeta-potential (mV) for AmB formulation at low (□) and high P (●)**. At high P, formulations are more loosely packed and efficiently deliver the aggregated drug to the fungus at low zeta-potentials. On the other hand, at low P, formulations are more tightly packed and efficiently deliver the monomeric drug (solubilized at the edges of the bilayer fragments) to the fungus at high zeta-potentials. Both formulations lead to zero % of fungus viability, a situation that cannot be achieved for cationic components of the particles in separate.

Alternatively, PDDA was replaced by PL (Table 2). At high P, the effect of increasing PL molecular weight was an increase in size, an increase in zeta-potential and a decrease of % of cell viability (Table 2). Table 1 summarized the different properties of assemblies at low and high P. One should notice that coverage of a drug particle with a thin DODAB layer led to a positive zeta-potential of only 9 mV. CMC was slightly attracted to the covered particle producing a looser assembly than the one obtained with CMC coverage of DODAB BF, where electrostatic attraction is due to a higher zeta-potential on the bilayer fragments, typically 41 mV. The particles are loosely or tightly packed depending on the electrostatic attraction between oppositely charged components (cationic layer and CMC) depicted from zeta-potentials. This certainly made a large difference for occasion of drug delivery to the fungus cell. Having a loose or a more tightly packed assembly originated considerable differences in the profile of cell viability as a function of zeta-potential (Figure 10). For the less tightly packed drug particles at high P, drug delivery was more efficient leading to drug release and cell death at lower zeta-potentials (Figure 10). The reason for this high efficiency at low zeta-potential is associated both with the high P, meaning high drug dose, and with the loosely packed nanoparticle assembly.

Fungizon (AmB in deoxycholate) and DODAB BF/AmB (formulation at low P) were previously evaluated in mice with systemic candidiasis [21]. Both formulations yielded equivalent therapeutic results. However, DODAB BF/AmB was better from the point of view of reduced nephrotoxicity [22]. Furthermore, cationic surfactants and polymers have an effect on integrity of red blood cells [28]. Therefore, similar studies should be performed for the formulations described in this paper.

## Conclusion

Optimal colloid stability and maximal fungicidal activity of monomeric or aggregated AmB in cationic lipid was achieved for cationic formulations at low or high drug to lipid molar proportions. At 0.005 mM drug, 1 mM DODAB, 1 mg/mL CMC and ≥ 5 mg/mL PDDA, monomeric AmB was found in DODAB BF enveloped by the two oppositely charged polyelectrolytes yielding 0% *C. albicans *viability. At 0.05 mM drug, 0.1 mM DODAB, 0.1 mg/mL CMC and PDDA ≥ 2 mg/mL, AmB/DODAB BF/CMC/PDDA assembly contained AmB in the aggregated state forming drug particles sequentially covered by DODAB BF, CMC and PDDA yielding also 0% fungus viability. The less tightly packed assembly turned out to be the one at high P, and high drug concentration which easily delivered the drug to cells at the lower zeta-potentials. The more tightly packed assembly was the one at low P, delivering drug to cells at higher zeta-potentials and lower drug concentration. *In vitro *both types of AmB formulations yielded complete fungicidal effect against *Candida albicans *(1 × 10^6 ^cfu/mL) representing good candidates to further tests *in vivo*.

## Methods

### Drug, lipid, polyelectrolytes and microorganism

Dioctadecyldimethylammonium bromide (DODAB), 99.9% pure was obtained from Sigma Co. (St. Louis, MO, USA). Carboxymethyl cellulose sodium salt (CMC) with a nominal mean degree of substitution (DS) of 0.60–0.95, poly(diallyldimethylammonium chloride) (PDDA) with M_v _100,000–200,000 and polylysines (PL) were obtained from Sigma (Steinheim, Germany) and used without further purification. Amphotericin B (AmB, batch 008000336) was purchased from Bristol-Myers Squibb (Brazil) and was initially prepared as a 1 g/L stock solution in DMSO/methanol 1:1. *Candida albicans *ATCC 90028 was purchased from American Type Culture Collection (ATCC) and reactivated in Sabouraud liquid broth 4% before plating for incubation at 37°C/24 h. In order to prepare fungal cell suspension for antifungal activity assays, three to four colonies were picked from the plate and washed twice either in isotonic glucose phosphate buffer (IGP; 1 mM potassium phosphate buffer, pH 7.0, supplemented with 287 mM glucose as an osmoprotectant) [39,40] or in Milli-Q water by centrifugation (3000 rpm/10 minutes), pelleting and resuspension. The final fungal cell suspension was prepared by adjusting the inoculum to 2 × 10^7 ^cfu/mL and then diluting by a factor of 1:10 either in IGP or in Milli-Q water yielding 2 × 10^6 ^cfu/mL.

### Preparation of lipid dispersion and analytical determination of lipid concentration

DODAB was dispersed in water or IGP buffer, using a titanium macrotip probe [41]. The macrotip probe was powered by ultrasound at a nominal output of 90 W (10 minutes, 70°C) to disperse 32 mg of DODAB powder in 25 mL water solution. The dispersion was centrifuged (60 minutes, 10000 g, 4°C) in order to eliminate residual titanium ejected from the macrotip. This procedure dispersed the amphiphile powder in aqueous solution using a high-energy input, which not only produced bilayer vesicles but also disrupted these vesicles, thereby generating open BF [29,41]. Analytical concentration of DODAB was determined by halide microtitration [42] and adjusted to 2 mM.

### Determination of zeta-average diameter and zeta-potential for dispersions

Stock solutions of AmB were prepared at 1 mg/mL in 1:1 DMSO/methanol. Stock solutions of PDDA, CMC and PL were prepared at 20 mg/mL and diluted in the final dispersion to yield the desired final concentration. The stock solution of AmB (1 mg/mL) was added to DODAB BF dispersions to yield low and high drug to lipid molar proportions (P). At low P, dispersions contained final concentrations of drug, DODAB, CMC and PDDA equal to 0.005 mM (5 micrograms/mL), 1 mM (631 micrograms/mL), 0.01–2.00 mg/mL and 0.01–10.00 mg/mL, respectively. Firstly, DODAB BF and drug were allowed to interact for 10 minutes. Thereafter, CMC was added and allowed to interact for 20 minutes before adding PDDA, which was also allowed to interact for 20 minutes, before determining zeta-average diameter and zeta-potentials. At high P, a similar procedure was done this time at final concentrations of drug, DODAB, CMC and PDDA equal to 0.050 mM (50 micrograms/mL), 0.1 mM (63.1 micrograms/mL), 0.01–2.00 mg/mL and 0.01–10.00 mg/mL, respectively. At high P, drug particles were obtained at 0.050 mM AmB in IGP buffer yielding particles with 75 nm zeta-average diameter and -27 mV zeta-potential [35]. These drug particles were firstly covered by DODAB BF and then wrapped by the polyelectrolytes over the quoted range of concentrations. Sizes and zeta-potentials were determined by means of a ZetaPlus Zeta-Potential Analyser (Brookhaven Instruments Corporation, Holtsville, NY, USA) equipped with a 570 nm laser and dynamic light scattering at 90° for particle sizing [43]. The zeta-average diameters referred to in this work from now on should be understood as the mean hydrodynamic diameters *D*_z_. Zeta-potentials (ζ) were determined from the electrophoretic mobility μ and Smoluchowski's equation, ζ = μη/ε, where η and ε are medium viscosity and dielectric constant, respectively. All *D*_z _and ζ were obtained at 25°C, 1 h after mixing.

### Optical spectra and aggregation state of AmB in the formulations

UV-visible optical spectra (280–450 nm) for characterization of AmB aggregation state were obtained in the double-beam mode by means of a Hitachi U-2000 Spectrophotometer against a blank of DODAB BF or DODAB BF/CMC (without drug), to separate light scattered by the dispersions from light absorption by the drug. All spectra were obtained at room temperature (25°C) at about 20 minutes after mixing DODAB BF and AmB at low or high drug to lipid P or after adding CMC to DODAB BF/drug assemblies.

### Determination of cell viability for C. albicans ATCC 90028 as a function of polyelectrolytes concentration at low and high drug to lipid molar proportion (P)

At low or high P, DODAB/drug assemblies were wrapped by two layers of oppositely charged polyelectrolytes so that cfu were counted as a function of CMC and/or PDDA concentrations at 1 h of interaction time between *C. albicans *(1 × 10^6 ^cfu/mL) and formulations. Plating on agar plates for cfu counts was performed by taking 0.1 mL of a 1000-fold dilution in Milli-Q water of the mixtures. After spreading, plates were incubated for 2 days at 37°C. CFU counts were made using a colony counter. At low P, final DMSO/methanol concentration is 0.5% whereas at high P it is 5%. No effect of the solvent mixture at 0.5% on cells viability was previously detected [25]. For further studies *in vivo *and at high P, it will be advisable to perform a dialysis step for the cationic nanoparticles aiming at complete elimination of the toxic solvent mixture.

## Competing interests

The authors declare that they have no competing interests.

## Authors' contributions

DBV did all of the experiments and data analysis in the laboratory, AMCR coordinated experiments, provided important advice for the experiments and financial support. Both authors read and approved the final manuscript.
